# Gut Microbiota Profile in Pediatric Patients With Inflammatory Bowel Disease: A Systematic Review

**DOI:** 10.3389/fped.2021.626232

**Published:** 2021-02-02

**Authors:** Xiaojun Zhuang, Caiguang Liu, Shukai Zhan, Zhenyi Tian, Na Li, Ren Mao, Zhirong Zeng, Minhu Chen

**Affiliations:** Department of Gastroenterology, The First Affiliated Hospital, Sun Yat-sen University, Guangzhou, China

**Keywords:** inflammatory bowel disease, Crohn's disease, ulcerative colitis, pediatric, gut microbiota

## Abstract

**Background and Aim:** Accumulating evidence have implicated gut microbiota alterations in pediatric and adult patients with inflammatory bowel disease (IBD); however, the results of different studies are often inconsistent and even contradictory. It is believed that early changes in new-onset and treatment-naïve pediatric patients are more informative. We performed a systematic review to investigate the gut microbiota profiles in pediatric IBD and identify specific microbiota biomarkers associated with this disorder.

**Methods:** Electronic databases were searched from inception to 31 July 2020 for studies that observed gut microbiota alterations in pediatric patients with IBD. Study quality was assessed using the Newcastle–Ottawa scale.

**Results:** A total of 41 original studies investigating gut microbiota profiles in pediatric patients with IBD were included in this review. Several studies have reported a decrease in α-diversity and an overall difference in β-diversity. Although no specific gut microbiota alterations were consistently reported, a gain in *Enterococcus* and a significant decrease in *Anaerostipes, Blautia, Coprococcus, Faecalibacterium, Roseburia, Ruminococcus*, and *Lachnospira* were found in the majority of the included articles. Moreover, there is insufficient data to show specific microbiota bacteria associated with disease activity, location, and behavior in pediatric IBD.

**Conclusions:** This systematic review identified evidence for differences in the abundance of some bacteria in pediatric patients with IBD when compared to patients without IBD; however, no clear overall conclusion could be drawn from the included studies due to inconsistent results and heterogeneous methodologies. Further studies with large samples that follow more rigorous and standardized methodologies are needed.

## Introduction

Inflammatory bowel disease (IBD) includes a group of complicated, long-lasting, and relapsing-remitting inflammatory disorders, of which ulcerative colitis (UC) and Crohn's disease (CD) are the two most prevalent subtypes (1, 2). The incidence of IBD has increased steadily in the Western world, but the rising incidence identified in newly industrialized countries is expected to continue to climb (3). IBD often occurs in young adulthood, but pediatric patients are also increasingly affected with 4% of patients with IBD younger than 5 years of age and 18% younger than 10 years of age (4). The epidemiologic characteristics, clinical presentation, and natural history differ depending on the age-of-diagnosis of IBD, while the potential mechanisms for disease heterogeneity remain poorly understood (5). Approximately 25% of patients with IBD demonstrate clinical manifestations during adolescence and young adulthood, and may experience lifelong disease courses with potential negative effects on growth, development, psychosocial function, and overall well-being (6). In addition, children with IBD exhibit a more severe course, especially those with a very early onset. Although the detailed pathogenesis of IBD remains unexplained, genetic predisposition, dietary patterns, inappropriate immune responses, and environmental factors have been reported to be closely associated with IBD (7–10). Though genetic factors seem to play a great role in patients with pediatric-onset IBD, environmental and microbial factors show a more prominent role in the occurrence and development of this disease in pediatric patients (11–13).

The human gut microbiota colonization is dynamic during early life and development owing to numerous factors, which may influence multiple physiological and pathophysiological processes throughout a patient's life (14, 15). The disruption of the ecological balance of gut microbiota composition and bacterial function triggers an aberrant immune response, leading to chronic intestinal inflammation. With advances in multi-omics methods, the study of gut microbial composition and function alterations in various diseases has emerged as a new potential area of clinical significance (16, 17). Gut microbiota imbalances identified in pediatric and adult patients from clinical and experimental studies over the past decade have shed light on the pathogenesis of IBD; however, the results differ and are sometimes contradictory owing to small sample sizes or heterogeneous methods (18–20). A growing number of studies have reported that the early human gut microbiota composition/development may affect adult health conditions, indicating that the gut microbiota profile in pediatric-onset IBD patients is associated with long-term consequences (14). However, comprehensive reviews of gut microbiota alterations in pediatric patients with IBD are rare, which may limit the development of new therapeutic options and the improvement of existing therapies for pediatric IBD (21, 22).

In order to facilitate the use of gut microbiota-targeted diagnoses and treatment methods in pediatric-onset IBD, a better understanding of what microbiota are associated with this disorder and how they affect the occurrence and development of IBD is urgently needed. However, most studies paid attention to the alterations of intestinal bacteria rather than viruses, protozoa and helminths. Thus, the aim of this systematic review was to investigate and describe the current findings relating to altered gut bacteria composition in pediatric patients with IBD, and to summarize specific bacteria taxa profiles associated with this disorder that distinguish pediatric IBD from adult IBD.

## Methods

### Search Protocol

The protocol for this systematic review is registered on the International Prospective Register of Systematic Reviews (PROSPERO) and the Preferred Reporting Items for Systematic Review and Meta-Analyses (PRISMA) checklist was used as a guideline. A comprehensive search was performed on public databases, including PubMed, Web of Science, Embase, Scopus, and the Cochrane Library (last search: 31 July 2020), with no date or language restrictions. The MeSH terms and free-text word combinations used in the search included: “Crohn's disease,” “CD,” “Ulcerative colitis,” “UC,” “Inflammatory bowel disease,” “IBD,” “microbiota,” “microbiome,” “microflora,” “bacterial flora,” “bacteria,” “children,” and “pediatric.”

### Eligibility Criteria and Study Selection

Articles were selected based on the following criteria: original studies performed in pediatric patients with IBD, CD, or UC diagnosis; assessment of the microbial communities of pediatric patients from different samples; data for bacterial profiles at different taxonomic levels provided; and the inclusion of a control group to which the gut microbiota was compared. Articles obtained from the initial literature searches were combined, and the duplicates were automatically removed. Titles and abstracts were screened by two independent investigators in the first round of the screening process. Next, potential studies were arranged for whole-paper reading, and their accompanying references were screened to identify additional eligible articles. Any discrepancies between the two investigators were resolved through consensus-based discussion, and a third reviewer was involved if necessary.

### Data Extraction and Quality Assessment

Data extracted from the included studies included demographic and clinical characteristics (such as year of publication, country, gender, IBD subtype, number of patients, disease activity, and treatment status), bacterial richness and diversity, taxonomic bacterial composition, and methodology of microbiota collection and evaluation (such as type of specimen, DNA extraction method, microbiota assessment techniques, reference database, and multiple comparison correction).

The quality of the included case-control studies was evaluated using the Newcastle-Ottawa Scale (NOS) (23). NOS contains three domains: selection (adequacy of case definition, representativeness of the cases, selection of controls, and definition of controls); comparability (comparability of baseline characteristics); and exposure (ascertainment of exposure, consistent method of ascertainment for cases and controls, and attrition rate). A final score of 0–3 indicated low quality, 4–6 indicated medium quality, and 7–9 indicated high quality.

## Results

### Study Selection and Quality Assessment

**Figure 1:**
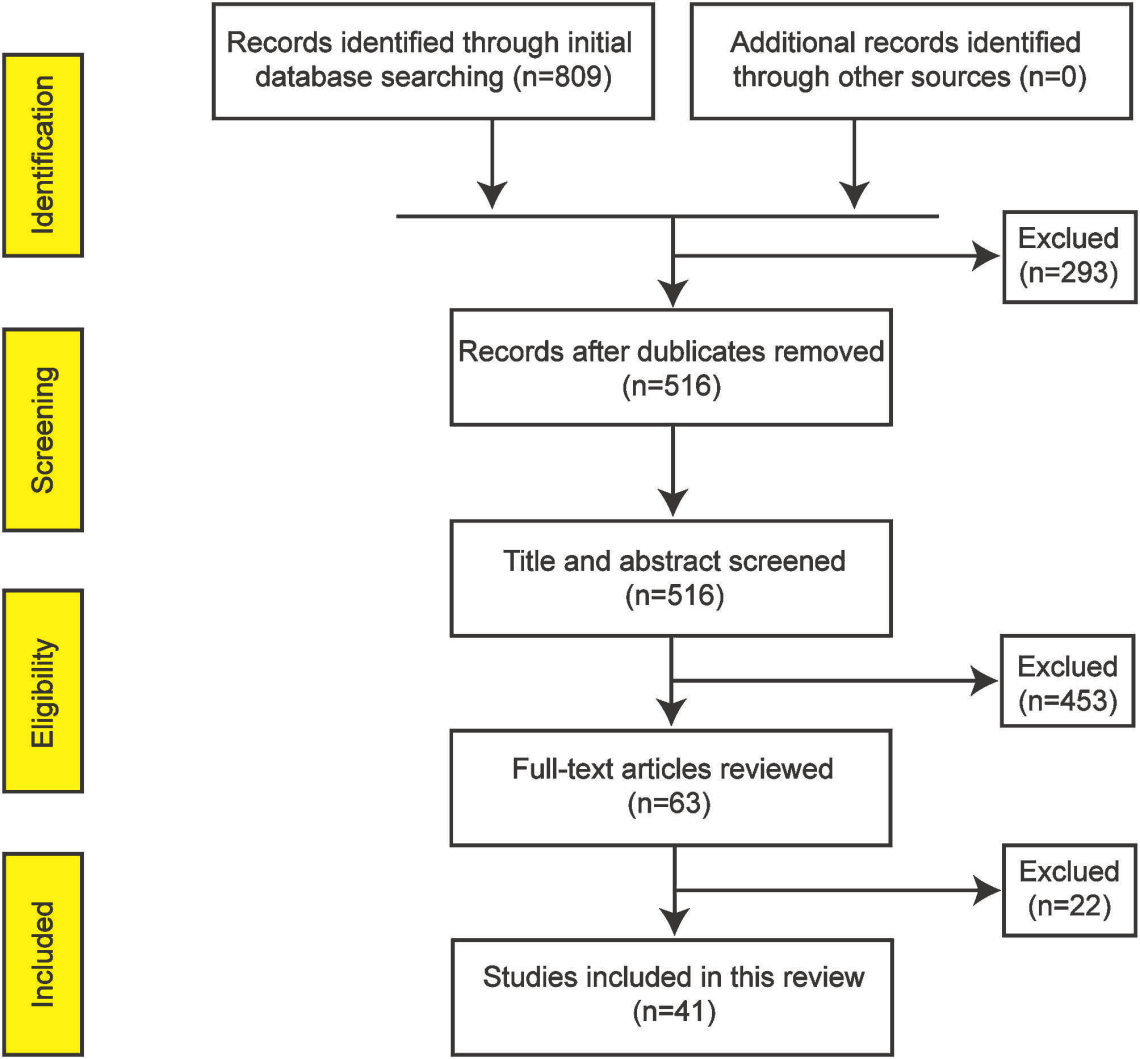
PRISMA flow diagram summarizing the studies identified during selection process.

The initial database search yielded 809 citations. The removal of duplicate articles resulted in 516 unique records. Subsequently, 63 studies were assigned to a whole-paper review after a secondary screening based on titles and abstracts. Finally, 41 original articles investigating the gut microbiota profile in pediatric patients with IBD were included for further review according to the inclusion criteria (Figure 1). As shown in Supplementary Table 1, all included studies received a moderate NOS score between 6 and 8. Studies that received a score of 8 strictly controlled multiple variables (in contrast to only controlling age). Ten studies received a score of 6 (moderate) due to control groups consisting of pediatric patients with functional gastrointestinal disorders, which is not representative of the community, or control groups being older or younger than IBD groups.

### Characteristics of Studies Investigating IBD

Four studies reported data in patients with UC, 19 studies evaluated patients with CD, 11 studies included both CD and UC, and 7 studies assessed patients with IBD and did not differentiate subtypes (Table 1). Nineteen studies recruited patients with active disease, 11 with the combination of active and remissive disease, 10 with unreported disease activity, and only one study investigated patients in remission. Geographically, the included studies were performed in Asia (China, Japan, and Saudi Arabia), Europe (UK, Sweden, Poland, Australia, Italy, Czech Republic, Croatia, Norway, Denmark, Germany, and Finland), and North America (USA and Canada). This review included 2,188 pediatric patients with IBD (59.0% males) and 1,233 controls (51.8% males) without IBD. Non-related participants were chosen as controls in most of the included studies, while three studies compared patients to their siblings, and two compared cases to a control group consisting of relatives and non-relatives of patients. The majority of studies investigated patients that received medication, whereas 14 studies explored the treatment-naïve microbiome in new-onset patients.

**Table 1:** Characteristics of included studies investigating gut microbiota profiles in pediatric patients with IBD and controls.

				**Patients**				**Controls**	
**First author**	**Publication year**	**Country**	**Age/year**	**Disease type/No**.	**Disease activity**	**Male/No**.	**Treatment-naïve**	**Control type/No**.	**Male/ No**.
Nusbaum	2018	USA	4–17	UC/7	Active	–	–	Health relatives/7	–
Sjoberg	2017	Sweden	9–16	UC/8	–	5	Yes	Health controls/8	–
Iwasawa	2016	Japan	10–22	UC/16	Active + Remissive	7	No	Health controls/23	10
Alipour	2016	Canada	4–17	UC/10	Active	2	No	Health controls/12	7
Shah	2016	USA	5–17	UC/10	–	4	Yes	Health controls/13	4
Michail	2012	USA, Canada	2–18	UC/27	Active	17	No	Health controls/26	14
Xue	2020	China	8.92 ± 4.80	CD/17	Active	11	No	Health controls/26	17
Duplaga	2020	Poland	12.27 ± 3.80	CD/18	Active	11	No	Health controls/18	8
Tang	2020	China	1.6–15.9	CD/31	Active	18	Yes	Health controls/12	8
Duplaga	2019	Poland	12.62 ± 3.53	CD/64	Active	39	Yes	Health controls/18	8
Kansal	2019	Australia	3.37–18.01	CD/138	–	87	No	Health controls/66	32
Haberman	2018	USA	≤ 16	CD/254	Active + Remissive	158	No	Health controls/50	31
Wang	2017	China	4–17	CD/11	Active	4	No	Health controls/16	–
Mouzan	2018	Saudi Arabia	7.3–17.8	CD/17	–	11	No	Health controls/18	12
Ijaz	2017	UK	10.9–13.8	CD/19	Active + Remissive	13	No	Related and unrelated controls/31	17
Assa	2016	Canada	11.25–16	CD/10	Active	6	Yes	Health controls/15	10
Mottawea	2016	Canada	11.75–17	CD/94	Active + Remissive	60	Yes	Health controls/63	30
Wang	2016	USA	2–20	CD/24	–	15	Yes	Health controls/63	30
Lewis	2015	USA, Canada	10.1–15.5	CD/85	Active	51	No	Health controls/26	14
Quince	2015	UK	6.9–14.7	CD/23	Active	13	No	Health controls/21	12
Haberman	2014	USA	12.2 ± 3.1	CD/243	Active + Remissive	146	No	Health controls/43	28
Gevers	2014	USA	3–17	CD/447	Active + Remissive	277	No	Health controls/221	115
Gerasimidis	2014	UK	12.7 ± 3	CD/15	Active	10	No	Health controls/21	12
Argenio	2013	Italy	15	CD/1	Active	1	No	Health controls/1	1
Kellermayer	2012	Czech Republic	8.11–17.3	CD/15	–	7	Yes	Controls without CD/9	5
Kaakoush	2012	Australia	11.6 ± 2.5	CD /19	Active + Remissive	12	Yes	Health controls/21	13
Leach	2008	Australia	10.2 ± 4.5	CD/6	Active	4	No	Health controls/7	3
Sila	2019	Croatia	14.8 ± 0.65	CD/13; UC/6	Active	12	Yes	Related and unrelated controls/39	16
Olbjørn	2019	Norway	0.74–17.9	CD/80; UC/27	Active + Remissive	43	Yes	Health controls/75	34
Malham	2019	Denmark	12.2–16.6	CD/77; UC/58	Active + Remissive	75	No	Health controls/34	17
Fernandes	2019	USA	12.2 ± 3.3	CD/7; UC/5	-	8	No	Health controls/12	5
Zhang	2018	Canada	12.3–16.1	CD/25; UC/22	Active	27	Yes	Health controls/24	10
Knoll	2016	Germany	14 ± 2.0	CD/6; UC/6	Active + Remissive	5	No	Health relatives/12	6
Shaw	2016	USA	≤ 17	CD/15; UC/4	Active	–	Yes	Health controls/10	–
Jacobs	2016	USA	13.6–19.7	CD/5; UC/1	–	5	No	Health relatives/21	10
Maukonen	2015	Finland	7–19	CD/10; UC/12	Active	–	No	Health controls/8	–
Hourigan	2015	USA	6–17	IBD/5	Active	–	No	Health controls/8	–
Nwosu	2013	Norway	11.3 ±0.4	CD/27; UC/16	–	22	Yes	Health controls/30	9
Aomatsu	2012	Japan	7.2–18	CD/10; UC/14	Remissive	10	No	Health controls/27	12
Hansen	2012	UK	6.3–16.3	CD/29; UC/13	–	29	No	Health controls/42	33
Papa	2012	USA	3–24	CD/23; UC/43	Active + Remissive	34	No	Health controls/24	10
Total				2188				1233	

*“–”, No information provided*.

### Sample Handling and Microbiome Assessment Methods

The sample handling and data analysis from individual studies are described in Table 2. Overall, 28 studies observed the gut microbiome from stool, 9 studies evaluated intestinal tissue from different segments, 2 studies used both stool and mucosal tissue, 1 study analyzed duodenal fluid, and 1 study investigated the mucosal–luminal interface aspirate. Most studies stored samples at either −70 or −80°C, after collection while seven studies did not provide information on sample storage. More than half of the studies implemented commercial spin column-based extraction kits according to the manufacturer's protocol for DNA extraction. Thirty-one studies assessed the gut microbiota by 16S rRNA gene sequencing targeting different variable regions and two used metagenome sequencing, while the remaining studies employed terminal restriction fragment length polymorphism, denaturing gradient gel electrophoresis, temporal temperature gradient gel electrophoresis, and GA-Map™ technology. Taxonomy was assessed using a variety of reference databases, most commonly Greengenes, RDP, or SILVA. Furthermore, approximately half of the studies (58.5%) performed multiple comparison tests when comparing gut microbiota alterations between IBD patients and controls.

**Table 2:** Samples collection and handling methodology of included studies.

**First author**	**Sample**	**Sample storage**	**DNA extraction**	**Sequencing technique/target**	**Reference database**	**Multiple comparison correction**
Nusbaum 2018	Stool	−80°C	Qiagen Dneasy DNA Extraction Kit	Illumina MiSeq/16S V4	GreenGenes	No
Sjoberg 2017	Duodenal fluid	−80°C	QIAamp DNA Stool Mini Kit	Pyrosequencing/16S V1–V3	RDP	No
Iwasawa 2016	Stool	NR	–	Pyrosequencing/16S V1–V2	–	No
Alipour 2016	Ileal biopsy	Snap-frozen	–	Illumina MiSeq/16S V3–V4	RDP	Yes
Shah 2016	Colonic biopsy	NR	–	Illumina MiSeq/16S V4–V6	GreenGenes	Yes
Michail 2012	Stool	−70°C	ZR Fecal DNA Kit	Microarray hybridization/16S	–	Yes
Xue 2020	Stool	−80°C	FastDNA SPIN Kit	Illumina MiSeq/16S V3–V4	–	Yes
Duplaga 2020	Stool	−70°C	–	Next-generation sequencing/16S V3–V4	GreenGenes	Yes
Tang 2020	Stool	−80°C	QIAamp DNA Stool Mini Kit	Illumina MiSeq/16S V3–V4	–	No
Duplaga 2019	Stool	−80°C	Genomic Mini AX Stool Spin Kit	Illumina MiSeq/16S V3–V4	GreenGenes	Yes
Kansal 2019	Ileocolonic biopsy	−70°C	QIAGEN AllPrep DNA/RNA Kit	Illumina MiSeq/16S V2	SILVA	Yes
Haberman 2018	Ileal biopsy	−80°C	Qiagen AllPrep RNA/DNA Mini Kit	Illumina MiSeq/16S V4	GreenGenes	Yes
Wang 2017	Stool	−80°C	QIAamp DNA Stool Mini Kit	Illumina MiSeq/16S V3–V4	GreenGenes	Yes
Mouzan 2018	Stool	−80°C	Mobio Powersoil Kit	Illumina MiSeq/16S V4	RDP	Yes
Ijaz 2017	Stool	NR	–	Illumina MiSeq/16S V4	RDP	Yes
Assa 2016	Ileal biopsy	−80°C	FastDNA SPIN Kit	Illumina HiSeq/16S V6	GreenGenes	Yes
Mottawea 2016	Mucosal-luminal interface	−80°C	Fast DNA Spin Kit	Illumina MiSeq/16S V6	GreenGenes	Yes
Wang 2016	Ileal biopsy + Stool	−80°C	QIAGEN Stool DNA Kit	Pyrosequencing/16S V1–V3	GreenGenes	Yes
Lewis 2015	Stool	−80°C	MoBio PowerSoil Kit	Illumina HiSeq/Metagenome	NCBI	Yes
Quince 2015	Stool	NR	–	Illumina MiSeq/16S V4	SILVA	Yes
Haberman 2014	Ileal biopsy	NR	–	Illumina MiSeq/16S V4	GreenGenes	Yes
Gevers 2014	Mucosal biopsy + Stool	−80°C	Qiagen AllPrep Mini Kit	Illumina MiSeq/16S V4	–	No
Gerasimidis 2014	Stool	−70°C	–	TTGE/16S V6–V8	–	No
Argenio 2013	Ileal biopsy	NR	–	Next-generation sequencing/16S V4–V6	–	No
Kellermayer 2012	Stool	−80°C	QIAamp DNA Mini Kit	Pyrosequencing/16S V1–V3	RDP	Yes
Kaakoush 2012	Stool	–	ISOLATE fecal DNA Kit	Pyrosequencing/V1–V3	GreenGenes	Yes
Leach 2008	Stool	−80°C	QIAamp DNA stool mini Kit	DGGE /16S	–	No
Sila 2019	Stool	−80°C	Quick-DNATM Fecal/Soil Microbe Miniprep Kit	T-RFLP/16S	RDP	No
Olbjørn 2019	Stool	−80°C	–	GA-Map/16S V3–V7	–	No
Malham 2019	Stool	−80°C	FastDNA SPIN Kit	Illumina HiSeq/16S V3–V4	RDP	Yes
Fernandes 2019	Stool	−80°C	–	Illumina MiSeq/V4	Greengenes	Yes
Zhang 2018	Mucosal biopsy	−80°C	–	Metaproteomics	NCBI	No
Knoll 2016	Stool	−80°C	–	Illumina HiSeq/Metagenomic	–	Yes
Shaw 2016	Stool	−80°C	–	Illumina MiSeq/16S V4	GreenGenes	Yes
Jacobs 2016	Stool	−80°C	MoBio PowerSoil DNA Isolation Kit	Illumina HiSeq/16S V4	GreenGenes	Yes
Maukonen 2015	Stool	−70°C	–	PCR-DGGE/16S V6–V8	RDP	No
Hourigan 2015	Stool	−80°C	MoBio PowerSoil DNA Isolation Kit	Illumina MiSeq/16S V4	GreenGenes	No
Nwosu 2013	Stool	−80°C	–	Mixed Sequencin/V3–V9	RDP	No
Aomatsu 2012	Stool	NR	MagaZorb DNA Common Kit	T-RFLP/16S V3–V7	InfoCom T-RFLP	No
Hansen 2012	Ileal biopsy	−80°C	Qiagen QIAamp Mini Kit	Pyrosequencing/16S V3–V6	–	No
Papa 2012	Stool	−80°C	QIAamp DNA Stool Mini Kit	Pyrosequencing/16S V3–V5	RDP	No

*“–”, No information provided*.

### Characteristics of Gut Microbiota in Pediatric CD

Thirty studies investigated gut microbiota profiles from pediatric patients with CD, and a majority reported microbial data from fecal samples. The remaining studies received microbial data from biopsy specimens, mucosal–luminal interface aspirate, and duodenal fluid. As shown in Table 3, the gut microbial communities of pediatric patients with CD were compared to controls, assessing α- and β-diversity as well as individual bacterial alterations at different taxonomic levels. The majority of studies reported that the richness and diversity (α-diversity) of gut microbiota in pediatric patients with CD were decreased and their gut microbiota β-diversity differed from controls. Intriguingly, these altered trends were observed to be more consistent in fecal microbiota than in mucosal microbiota. However, several studies found no difference in the α- or β-diversity of the gut microbiota between subjects with and without IBD. Consistent microbiota alterations at various taxonomic levels from fecal or mucosal samples in pediatric patients with CD had been highlighted (with a more comprehensive summary in Supplementary Table 2), though highly heterogeneous results existed in the included studies.

**Table 3:** Consistent gut microbiota alterations in pediatric patients with CD compared to controls at different taxonomies.

**References**	**(24)**	**(25)**	**(26)**	**(27)**	**(28)**	**(29)**	**(30)**	**(31)**	**(30)**	**(32)**	**(33)**	**(18)**	**(34)**	**(35)**	**(36)**	**(37)**	**(38)**	**(19)**	**(39)**	**(40)**	**(41)**	**(42)**	**(43)**	**(44)**	**(45)**	**(46)**	**(47)**	**(48)**	**(49)**	**(50)**	**Total**	
Sample	S	S	S	S	S	S	S	S	S	S	S	S	S	S	S	S	S	S	S	IM + S	M + S	ICM	IM	IM	IM	IM	IM	IM	MLA	DF	↑	↓
**α-dicersity**																																
Richness	↓	↓	↓	↓	↓	–	↓	↓	↓	↓	N	–	–	↓	↓	↓	↓	↓	–	–	↓	↓	↓	N	–	↓	N	↓	N	N	0	19
Diversity	↓	↓	↓	↓	↓	↓	↓	↓	↓	↓	N	–	–	↓	↓	↓	↓	↓	–	–	↓	N	–	N	–	↓	N	↓	N	–	0	18
**β-diversity**	D	D	D	D	D	D	D	D	D	–	D	N	–	N	–	D	–	–	–	D	D	D	D	N	D	–	N	N	N	D	D = 17	
**Order**																																
Bacteroidales					↓															↓	↓				↓						0	4
Bifidobacteriales	↓				↓															↓					↓						0	4
Clostridiales	↓															↓				↓	↓	↓	↓		↓						0	7
**Family**																																
Bifidobacteriaceae	↓				↓															↓	↓										0	4
Coriobacteriaceas	↓	↓															↓													↓	0	4
Enterobacteriacese					↑		↑									↑				↑	↑		↑			↑	↑		↑		9	0
Erysipelotrichaceae						↓														↓	↓				↓						0	4
Fusobacteriaceae						↑														↑	↑				↑						4	0
Gemellaceae		↑																		↑	↑				↑						4	0
Pasteurellaceae							↑													↑	↑				↑	↑					5	0
Ruminococcaceae	↓	↓			↓		↓		↓			↓								↓			↓	↑			↑				2	8
Veillonellaceae	↑																			↑	↑								↑		4	0
**Genus**																																
Alistipes			↓		↓			↓	↓																						0	4
Bilophila			↓		↓			↓	↓																						0	4
Clostridium		↓		↓		↓		↓	↓											↓				↓						↓	0	8
Enterococcus		↑	↑	↑	↑		↑	↑								↑		↑													8	0
Granulicatella	↑	↑	↑					↑																							4	0
Lachnospira			↓		↓				↓							↓				↓											0	5
Odoribacter			↓		↓			↓																↓							0	4
Parabacteroides			↓				↓	↓	↓											↓											0	5
Roseburia			↓	↓	↓	↓		↓	↓		↓	↓				↓						↓	↓						↓		0	12
Ruminococcus		↓	↓		↓	↓		↓	↓											↓											0	7
Streptococcus		↑	↑		↑	↓		↑	↑							↑								↓							6	2
Subdoligranulum			↓						↓		↓	↓																			0	4
Turicibacter					↓	↓			↓											↓			↓								0	5

*Only bacteria, for which all included studies have agreed on the direction of difference, are displayed in the table. “↑” = higher α diversity or bacteria are more abundant, in CD compared to control; “↓” = Lower α diversity or bacteria are less abundant, in CD compared to control; “D” = bacterial β-diversity differ between CD patients compared to controls; “N” = No difference in α diversity or β-diversity; “–” = no information. CD, Crohn's disease; S, Stool; IM, Ileal mucosa; ICM, Ileocolonic mucosa; MLA, Mucosal-luminal interface aspirate; DF, Duodenal fluid*.

At the phylum level, a decreased abundance of Actinobacteria and Bacteroidetes, and an increased abundance of Proteobacteria were reported in pediatric patients with CD, which is similar to the results of studies that assessed adult patients. However, these consistent results were reported in a very small number of studies. At the class level, no clear overall conclusion about gut microbiota alterations could be drawn from the included studies, but decreased amounts of Clostridia and increased amounts of Gammaproteobacteria were noted. Additionally, several studies have demonstrated a significant decrease in the orders Bacteroidales, Clostridiales, Bifidobacteriales, and Erysipelotrichales as well as an increase in Enterobacteriales and Fusobacteriales. In terms of lower taxonomic levels, previous studies have demonstrated inconclusive results of gut microbiota alterations in pediatric patients with CD. Even so, a decreased relative abundance of several genera belonging to the families Lachnospiraceae (*Anaerostipes, Blautia, Coprococcus, Lachnospira*, and *Roseburia*), Ruminococcaceae (*Anaerotruncus, Faecalibacterium*, and *Ruminococcus*), Clostridium, Holdemania, Odoribacter, Parabacteroides, and Turicibacter were reported in most included studies. In addition, the abundance of the genera *Actinomyces, Corynebacterium, Enterococcus, Escherichia, Fusobacterium, Granulicatella, Sutterella*, and *Veillonella* were assessed in several studies. However, some bacteria from duodenal fluid showed an opposite change trend when compared to feces-associated and mucosa-associated microbiota, with a decrease in Proteobacteria and Enterobacteriaceae, and an increase in the family Erysipelotrichaceae.

### Characteristics of Gut Microbiota in Pediatric UC

Fifteen studies evaluated the gut microbiota profile in pediatric patients with UC, with nine studies assessing feces-associated microbiota, five studies using mucosa-associated microbiota, and only one study analyzing duodenal fluid-associated microbiota. Nine of 15 UC studies showed a significant decrease in the α-diversity indexes and 5 studies revealed β-diversity differences between the microbiota of patients with UC, while the remaining study found no differences. A comprehensive list of gut microbiota alterations in pediatric patients with UC is shown in Table 4 (with a more comprehensive summary in Supplementary Table 3). Similarly, patients with UC tended to have a decreased abundance of bacteria belonging to the phylum Firmicutes, though this was only reported in two studies. In addition, the differences in Proteobacteria and Verrucomicrobiae were not consistent among the studies. Additionally, five studies reported decreased bacteria in the class Clostridia and three studies reported decreased bacteria in the order Clostridiales in pediatric patients with UC compared to controls. Bacteria in the family Lachnospiraceae were decreased in six studies, and bacteria in the family Enterobacteriacese was decreased in three studies. No clear overall conclusions could be drawn from the included studies regarding alterations at the genus level. Several studies have reported an increased abundance of *Haemophilus* and *Veillonella* as well as a reduced abundance of *Alistipes, Anaerostipes, Blautia, Oscillospira, Roseburia*, and *Ruminococcus*. The genera *Bacteroides, Bifidobacterium, Clostridium, Faecalibacterium*, and *Parabacteroides* showed no consistent changes in the included studies, which is distinct from the findings in pediatric patients with CD.

**Table 4:** Consistent gut microbiota alterations in pediatric patients with UC compared to controls at different taxonomies.

**References**	**(51)**	**(52)**	**(53)**	**(35)**	**(36)**	**(37)**	**(38)**	**(19)**	**(39)**	**(43)**	**(47)**	**(48)**	**(54)**	**(49)**	**(50)**	**Total**	
Sample	S	S	S	S	S	S	S	S	S	IM	IM	IM	CM	MLA	DF	↑	↓
**α-dicersity**																	
Richness	↓	↓	↓	↓	↓	↓	↓	N	–	–	↓	N	N	N	↓	0	9
Diversity	↓	↓	↓	↓	↓	↓	↓	N	–	–	↓	N	N	N	–	0	8
**β-diversity**	N	D	–	N	–	D	–	–	–	D	N	N	D	N	D	D = 5	
**Phylum**																	
Firmicutes			↓		↓											0	2
Proteobacteria			↑											↑		2	2
**Class**																	
Bacilli			↓												↓	0	2
Betaproteobacteria			↓												↓	0	2
Clostridia	↓		↓		↓					↓					↓	0	5
Erysipelotrichi			↓							↓						0	2
**Order**																	
Clostridiales					↓					↓			↓			0	3
**Family**																	
Bacteroidaceae					↑						↑					0	2
Coriobacteriaceas							↓								↓	0	2
Enterobacteriacese					↑	↑					↑					3	0
Lachnospiraceae	↓						↓			↓	↓		↓	↓		0	6
Rikenellaceae											↓		↓	↓		0	3
**Genus**																	
Alistipes						↓								↓		0	2
Anaerostipes	↓	↓														0	2
Enterococcus		↑				↑										2	0
Eubacterium		↓			↓											0	2
Haemophilus						↑		↑						↑		3	0
Oscillospira						↓							↓			0	2
Roseburia	↓	↓											↓			0	3
Ruminococcus		↓				↓							↓			0	3
Veillonella						↑								↑		2	0

*Only bacteria, for which all included studies have agreed on the direction of difference, are displayed in the table. “↑” = higher α diversity or bacteria are more abundant, in UC compared to control; “↓” = Lower α diversity or bacteria are less abundant, in UC compared to control; “D” = bacterial β-diversity differ between UC patients compared to controls; “N” = No difference in α diversity or β-diversity; “–” = no information*.

*UC, Ulcerative colitis; S, Stool; IM, Ileal mucosa; ICM, Ileocolonic mucosa; MLA, Mucosal-luminal interface aspirate; DF, Duodenal fluid*.

### Characteristics of Gut Microbiota in Pediatric IBD

In an attempt to define the specific gut microbiota profiles in pediatric patients with IBD, several studies have compared IBD cases with controls without IBD (Table 5). However, the evaluation of these studies is limited due to the different compositions of mucosal and luminal microbiota and small sample sizes. Eight studies aimed to identify specific gut microbiota alterations in pediatric patients with IBD using case-control studies, while no consistent and definitive conclusions could be drawn from the included studies (Table 5). Seven studies assessed intestinal microbiota from feces, one study evaluated mucosal–luminal interface aspirates, and one study analyzed duodenal fluid. All studies investigating feces-associated microbiota showed a significant decrease in the α-diversity in patients with IBD, however, only three studies reported that the microbiota differed between patients with IBD and controls. The most consistent finding among the studies is that the potentially harmful bacteria *Enterobacter* and *Escherichia* within the family Enterobacteriacese are increased in patients with IBD. Furthermore, increased amounts of *Actinobacillus, Prevotella, Streptococcus*, and *Veillonella* were found in pediatric patients with IBD, while the amounts of *Eubacterium, Lactobacillus*, and *Parabacteroides* were reported to be decreased. Conflicting results for changes in the genera *Bacillus, Bacteroides, Citrobacter*, and *Odoribacter* were reported. A more comprehensive list of gut microbiota alterations in pediatric patients with IBD at the phylum, class, order, family, and genus levels is presented in Supplementary Table 4.

**Table 5:** Consistent gut microbiota alterations in pediatric patients with IBD compared to controls at different taxonomies.

**References**	**(55)**	**(56)**	**(20)**	**(36)**	**(57)**	**(58)**	**(59)**	**(60)**	**(50)**	**Total**	
Sample	S	S	S	S	S	S	S	M	DF	↑	↓
**α-dicersity**											
Richness	↓	↓	–	↓	↓	↓	↓	–	–	0	6
Diversity	↓	↓	–	↓	↓	↓	↓	–	–	0	6
**β-diversity**	D	D	–	–	D	N	–	D	D	D = 5	
**Class**											
Clostridia				↓					↓	0	2
Gammaproteobacteria				↑			↑			2	0
**Order**											
Enterobacteriales				↑			↑			2	0
**Family**											
Enterobacteriacese						↑	↑			2	0
**Genus**											
Actinobacillus					↑	↑				2	0
Enterobacter	↑							↑		2	0
Escherichia					↑		↑	↑		3	0
Eubacterium		↓		↓						0	2
Haemophilus			↑		↑					2	0
Lactobacillus	↓								↓	0	2
Parabacteroides						↓			↓	0	2
Prevotella		↑						↑		2	0
Ruminococcus			↓		↓					0	2
Streptococcus	↑		↑					↑		3	0
Veillonella					↑	↑				2	0

*Only bacteria, for which all included studies have agreed on the direction of difference, are displayed in the table. “↑” = higher α diversity or bacteria are more abundant, in IBD compared to control; “↓” = Lower α diversity or bacteria are less abundant, in IBD compared to control; “D” = bacterial β-diversity differ between IBD patients compared to controls; “N” = No difference in β-diversity; “–” = no information*.

*IBD, Inflammatory bowel disease; S, Stool; MLA, Mucosal-luminal interface aspirate; DF, Duodenal fluid*.

### Effect of Naïve Treatment on Microbiota

The importance of the interaction between intestinal microbiota and specific medication used in IBD has been reported. The unique gut microbiota characteristics collected prior to treatment in new-onset cases may result in an early diagnosis and administration of specific medications in pediatric patients with IBD. Fourteen studies assessed the treatment-naïve microbiome in new-onset IBD, however, the reported changes were inconsistent. In patients with treatment-naïve CD, decreased amounts of *Clostridium, Coprococcus, Faecalibacterium, Roseburia, Ruminococcus*, and *Subdoligranulum*, and an increased amount of *Enterococcus* were reported. However, such alterations were also found in patients with CD who had been treated. Bacteria in the genus *Dialister* increased in pediatric patients with treatment-naïve CD and decreased in patients who received treatment prior to sample collection. There is insufficient data to determine the differences between the microbiota of patients with treatment-naïve UC and patients undergoing treatment for UC, due to the small number of studies and various locations of samples. A decrease in the abundance of bacteria in the family Lachnospiraceae and the genera *Clostridium, Roseburia*, and *Ruminococcus* were found consistently in pediatric patients with UC regardless of therapeutic interventions. *Haemophilus* bacteria increased in both non-treated and treated pediatric patients with UC. However, no signature of gut microbiota could be determined for pediatric patients with treatment-naïve UC.

### Comparing Pediatric Patients With Healthy Relatives

Although the gut microbiome changes throughout a patient's life, no systematic changes were found in patients with CD with different ages of diagnosis, suggesting that CD-associated dysbiosis is already established in younger CD patients. Five studies compared the gut microbiota of pediatric patients with IBD with those of healthy relatives and found significantly decreased microbial diversity with a distinct bacteria profile in pediatric patients with IBD. However, no consistent conclusions could be drawn. The group of healthy relatives had a significantly higher microbiota diversity richness and evenness than pediatric patients with IBD in some studies. Nevertheless, three studies reported that the relatives of pediatric patients with IBD had a distinct bacterial composition that differed from non-related controls, rather than a characteristically altered gut microbiota. Jacobs et al. reported that healthy first-degree relatives of pediatric patients with IBD may have a fecal microbial and metabolomic profile with a pre-disease susceptibility state or subclinical inflammation, which may be found in patients with quiescent IBD, suggesting the potential risk of this disease. In addition, several studies reported that healthy relatives might be suitable donors for fecal microbiota transplantation as they share genetic and environmental factors with the patients.

### Differences in Feces-Associated and Mucosa-Associated Microbiota

The majority of studies included in this report used fecal samples to assess the gut microbial diversity and composition in pediatric patients with IBD, which is a taxonomically distinct mucosal microbiome. Due to the high availability of feces-associated microbiota, consistent findings were found between the studies, though the mucosa-associated microbiota may be more likely to play a role in IBD. For pediatric patients with CD, the alterations in feces-associated microbiota differed from those in mucosa-associated microbiota (such as decreased amounts of *Alistipes, Bacteroides, Bilophila, Dialister, Faecalibacterium*, and *Subdoligranulum*, and an increased amount of *Enterococcus*). In addition, several taxa showed opposite trends between fecal samples and mucosal samples. For example, a low amount of bacteria in the family Porphyromonadaceae in mucosa-associated microbiota was reported and a high amount was reported in feces-associated microbiota. Moreover, an increased abundance of *Dialister* was found in the mucosal microbiome, while a decreased amount was reported in fecal samples. For pediatric patients with UC, similar observations of gut microbiota alterations were found in fecal and mucosal samples. An increase in the amount of bacteria in the family Enterobacteriaceae and a decrease in *Roseburia* and *Ruminococcus* belonging to the family Lachnospiraceae were reported in both the mucosal and fecal samples. However, there is insufficient data to show distinctive gut microbiota profiles in pediatric patients with UC due to the small number of studies.

### Differences in Eastern and Western Populations

Knowledge regarding the geographic and environmental factors (dietary lifestyle) that are relevant for shaping the gut microbiota has increased considerably in recent years. The previously reported dysbiosis in adult-onset IBD patients may be already established during the pediatric period. Most of the studies included in this report were conducted in Western countries, which may limit the possibility of determining differences between studies from the East and West. Twenty-five studies assessed the microbiota of Western patients with CD, and 5 studies included Eastern patients (3 studies from China, 1 from Japan, and 1 from Saudi Arabia). The gut microbiota alterations in pediatric patients with CD that are consistent between Eastern and Western patients include decreased amounts of *Alistipes, Anaerotruncus, Bilophila, Clostridium, Lachnospira, Parabacteroides, Roseburia, Ruminococcus*, and *Turicibacter*, and increased amounts of *Enterobacter* and *Enterococcus*. Thirteen studies assessed the microbiota of Western patients with UC, and only two studies included Eastern patients (both from Japan). The data from these studies were insufficient to evaluate consistent findings between Eastern and Western patients. In this review, gut microbiota alterations from Eastern patients were similar to that from Western pediatric patients, suggesting that conclusive results could not obtained when different geographical locations or dietary lifestyles are considered.

### Disease Activity Associated With Gut Microbiota Alterations

Not all included studies reported the disease activity of patients. Nineteen studies evaluated the gut microbiota in patients with active disease and 11 studies assessed the gut microbiota for patients with both active and remissive disease. A strong association between microbiota diversity and disease activity has been reported in several studies independent of the type of disease (CD or UC). Although no conclusive determinations can be made, a diminished richness of the taxa and alterations of several specific bacteria have been reported to be associated with disease activity. Kaakoush et al. reported that the detection frequencies of Bacteroidetes and Firmicutes correlated (positively and negatively, respectively) with the calculated pediatric CD activity index (PCDAI) scores of patients. However, findings from Haberman et al. demonstrated that PCDAI scores were positively associated with Gammaproteobacteria and Enterobacteriaceae and negatively associated with Clostridiales and Bacteroides. Furthermore, Mottawea et al. found that major short-chain fatty acids (SCFA) producers such as Lachnospiraceae, *Blautia, Roseburia, Ruminococcus, Clostridium*, and *Faecalibacterium* were negatively correlated with disease severity, while some H_2_S producers such as *Atopobium, Fusobacterium, Veillonella, Prevotella, Streptoccocus*, and *Leptotrichia* were positively correlated with disease severity. Additionally, Gevers et al. assessed that the microbial dysbiosis index showed a strong positive correlation with PCDAI, and the levels of *Fusobacterium* and *Haemophilus* were positively correlated with PCDAI, which was further confirmed in a study by Shaw et al. Maukonen et al. found that *Bacteroides* was the only bacterial group in which lower disease activity was associated with higher bacterial numbers in both UC and CD. In addition, Xue et al. found a significant correlation between PCDAI and Simplified Endoscopic Score for Crohn's Disease (SES-CD) scores and the dysbiosis indices (Lactobacillales, Micrococcales, Veillonellaceae, Clostridiales, and Selenomonadales). Overall, conclusive results of specific gut microbiota alterations associated with disease activity could not be determined due to inconsistent data and methods among the included studies.

### Disease Location Associated With Gut Microbiota Alterations

Generalized dysbiosis independent of gut location has been previously demonstrated in a number of studies. Despite the limitation of a small sample size, several studies have attempted to investigate the relationship between disease location and gut microbiota alterations. However, the results of these studies are inconsistent and sometimes contradictory. Olbjørn et al. found that patients with CD with upper gastrointestinal involvement had an increased abundance of *Veillonella* compared to patients without upper gastrointestinal lesions. Gevers et al. reported that ileal and rectal biopsies have similar discriminatory power for classifying diseases, regardless of the disease location. Haberman et al. reported a similar microbial shift in the ilea between patients with ileal CD (iCD) and colonic CD (cCD), irrespective of histologic involvement in cCD, showing a decrease in the amount of Lachnospiraceae, Bifidobacteriaceae, Clostridiales, and Erysipelotrichaceae and an increase in the amount of Veillonellaceae, Pasteurellaceae, Neisseriaceae, Gemellaceae, Fusobacteriaceae, and Enterobacteriaceae. In addition, no significant differences in the bacterial community between inflamed and uninflamed mucosa in pediatric patients with CD has been reported.

### Disease Behavior Associated With Gut Microbiota Alterations

Data regarding disease behavior and gut microbiota alterations was limited, as few studies have focused on this association. One pediatric study evaluated the relationship between disease behavior and gut microbiota alterations. Olbjørn et al. found that higher abundance of *Proteobacteria in* pediatric patients with CD means more likely to have complicated disease behavior (structuring or penetrating disease) as compared to patients with lower levels of these bacteria.

## Discussion

Identifying characteristic gut microbiota changes may provide insight into the role of the gut microbiome in the etiology and treatment methods of pediatric IBD. Several reviews attempting to evaluate the gut microbiota profiles in adult patients with IBD found that patients with IBD tend to have a reduced abundance of bacteria belonging to the Firmicutes phylum and an increased abundance of bacteria from the Proteobacteria phylum (61–63). However, there is no review that identifies specific bacteria that can distinguish adult and pediatric patients with IBD. Several studies have reported that the microbiota composition changes gradually with time, and that even though the gut microbiota begins to resemble the adult flora by 3 years of age, it is crucial to study the gut microbiota of pediatric patients with IBD (64, 65). Additionally, IBD is dynamic and little is known about the individual nature of microbiome dynamics in IBD from childhood through adulthood (66). In this systematic review, we firstly sought to provide a unique framework for understanding microbial dysbiosis and to summarize specific gut microbiota alterations in pediatric patients with IBD compared to controls. Collectively, no specific bacteria consistently differed between pediatric patients with IBD and controls in each of the included studies due to the heterogeneity of the type of samples, sample handling, and microbiome assessment methods of the included studies. Even though pediatric CD and UC have unique gut microbiota alterations, no bacteria consistently differed in these patients among the studies included in this review. Furthermore, there is insufficient data to evaluate the gut microbiota associated with disease activity, lesion location, or disease behavior in pediatric patients with IBD.

In the majority of the studies included in this review, a decreased microbial biodiversity with alterations in the composition of the gut microbiota community was observed in fecal and mucosal samples from pediatric patients with IBD. In addition, a number of differences were found between patients with IBD and controls when comparing the relative abundance of individual bacterial taxonomy, although no distinct bacteria were consistently identified in all of the included studies. Consistent alterations in gut microbiota were identified among studies that used fecal samples. Similar to previous studies investigating adult patients with IBD, pediatric patients were reported to have an increased amount of *Enterococcus* and a significant decrease in *Anaerostipes, Blautia, Coprococcus, Faecalibacterium, Roseburia, Ruminococcus*, and *Lachnospira*. These findings suggest that the previously reported dysbiosis in patients with adult-onset IBD may be established during the pediatric period. In recent years, there is accumulating evidence suggesting that alterations in the metabolites of the gut microbiota and specific bacterial metabolic pathways may help elucidate the precise cause-effect mechanistic relationships between gut microbiota and IBD, which may result in new discoveries regarding the pathogenesis and therapeutic methods for this disease (67–69). In the majority of studies, a decreased abundance of *Anaerostipes, Lachnospira, Roseburia, Faecalibacterium, Coprococcus, Ruminococcus*, and *Roseburia* bacteria were observed in pediatric patients with CD. These bacteria produce SCFA, which may be the primary source of energy for colonocytes, create an environment for proper intestinal colonization, and show immunomodulatory and anti-inflammatory properties (70, 71). SCFA play essential roles in maintaining the intestinal barrier function via promoting Treg cell development and enhancing mucus production from goblet cells as well as in inflammation-associated immunosuppression via regulating chemokine and cytokine production (72–75). Additionally, vanished SCFA-producing bacteria may favor a shift toward an inflammation-promoting microbiome, thereby enhancing host inflammation and worsening the condition of CD (28). A depletion of several *Clostridium* species that adhere to epithelial cells can mediate diverse effects on mucosal immunity via altering the differentiation of T-helper 17 cells and regulatory T cells (76, 77). Other bacteria that were found to be decreased in pediatric patients with CD include *Akkermansia*, which may play a role in the intestinal barrier function by providing crucial anti-inflammatory responses (78), *Alistipes* may act as a colitis-attenuating bacteria with unclear mechanisms (79), while *Parabacteroides* may have anti-inflammatory and epithelium-reinforcing capacities (80), *Turicibacter* may cause alterations in the bacterial diversity of the host and subsequent changes in steroid and lipid metabolism by interacting with serotonin (81). Still, more research is required to determine the mechanisms of bacteria involved in IBD.

*Faecalibacterium prausnitzii*, a potentially protective bacterium associated with disease activity, was found to be decreased in pediatric patients with IBD only in studies analyzing fecal sampling. Gram-negative pathobionts including *Enterobacteriaceae* species (*Enterobacter* and *Escherichaia*), *Enterococcus* and *Fusobacterium* were increased in pediatric patients with IBD, and have been reported to have the ability to adhere to and invade intestinal epithelial cells, leading to an inflammatory immune response (82–85). In addition, alterations in the abundance of H_2_S-producing bacteria (*Atopobium, Fusobacterium, Veillonella, Prevotella*, and *Streptoccocus*) suggest a possible role for these bacteria in the pathogenesis of CD (49). H_2_S, an important mediator of many physiological and pathological processes, can damage the gastrointestinal epithelium and exerts influence on the gut microbiota and on mucus and biofilm interactions in the context of intestinal inflammation (86, 87). Furthermore, depletion of butyrate-producing microbes from the colonic microbiota may dampen host H_2_S defense systems, which exacerbates inflammation and markedly alters the intestinal microbiota biofilm (88). In addition, alterations in gut microbiota with other microbial metabolites (such as bile acid and tryptophan metabolites) may have important and diverse effects on immune maturation, immune homoeostasis, host energy metabolism, and maintenance of mucosal integrity (67, 69, 89–93). Although the gut microbiota profile in pediatric patients with IBD showed similar patterns to adult patients with IBD, it is believed that gut microbiota characterisations of pediatric patients without extraneous influences of adult behaviors are likely to be more informative and useful for the treatment of pediatric IBD.

Changes in gut microbiota composition and function are thought to be associated with disease activity, disease behavior, and lesion location in patients with IBD. A systematic review showed that patients with active IBD had a lower abundance of *Clostridium coccoides, Clostridium leptum, Faecalibacterium prausnitzii*, and *Bifidobacterium* (94). Although no specific bacteria were consistently associated with disease activity of pediatric IBD in this review, a negative association between the activity of IBD and the abundance of Clostridiales was found in several studies. There are insufficient data to evaluate differences in gut microbiota associated with the location of lesions as most samples were collected from the ileum. Haberman et al. reported a persistent decrease in Lachnospiraceae, Bifidobacteriaceae, Clostridiales, and Erysipelotrichaceae in all forms of CD, with an increase in Veillonellaceae, Pasteurellaceae, Neisseriaceae, Gemellaceae, Fusobacteriaceae, and Enterobacteriaceae bacteria, independent of cecal involvement (43), which differs from previously reported results that *Enterococcus faecalis* is more abundant when CD is localized in ileum compared with the ileocolon (95). A prospective inception cohort study found that *Ruminococcus* is associated with stricturing complications and *Veillonella* is associated with penetrating complications (96). However, a similar relationship in pediatric patients was not reported in the studies included in this review.

Several studies have explored the association between oral microbiota and CD as oral manifestations are very common. Kelsen et al. reported that *Capnocytophaga, Rothia*, and *TM7* were more abundant in subgingival plaques samples of pediatric patients with CD than in healthy controls (97). Iwasawa found that the abundance of *Streptococcus* from salivary samples was significantly lower in patients with UC than in healthy controls (98). Nevertheless, data on specific oral-associated microbiota changes involved in pediatric IBD is limited.

There are some limitations to this study that warrant discussion. It is widely known that the composition and function of the human gut microbiome is shaped by multiple factors, including host genetics, geographic location, demographics, dietary patterns, and life behaviors, which may explain discrepancies between different studies (99–102). The methodologies of these studies differ according to the type of IBD (both CD and UC, only CD or only UC), age of control group (healthy minor or related siblings or adult), disease activity (active or inactive), sample type (feces, mucosal biopsy, mucosal–luminal interface aspirate, or duodenal fluid), and methods used for microbiota analysis (DNA extraction, sequencing methodology, or data analysis), which may also result in highly heterogeneous gut microbiota compositions between studies. Although it has long been proposed that an altered gut microbiome plays an important role in IBD, a direct causal relationship between dysbiosis and the disease has not been definitively established in humans. A comprehensive understanding of gut microbiota profiles in pediatric patients with new-onset IBD prior to therapeutic intervention is required to understand gut microbiota alterations associated with the occurrence and development of IBD and whether potential relationships exist between specific bacteria and disease subtype, activity, and location.

## Conclusion

Altered gut microbiota composition in pediatric patients with IBD are associated with this disorder. Moreover, the gut microbiota profiles of pediatric patients with IBD are similar to those of adult patients. In addition, specific gut microbiota is associated with disease characteristics (subtype, activity, and location), however, the studies included in this review varied widely in geography and methodology, leading to considerable heterogeneity in our findings. Future studies should consider bacteria species or strain levels with gut microbiota functions to elucidate the causative or correlative relationships between microbial changes and pediatric IBD. Furthermore, prospective, well-designed trials of adults and newly-diagnosed pediatric patients are required to reveal the typical gut microbiota changes associated with the onset of IBD. Determining specific gut microbiota associated with pediatric IBD may allow for the development of microbiome-targeted interventions, which may influence the natural course of disease or prevent the disease from developing. Future research using rigorous and standardized methodologies will lead to more consistent results.

## Data Availability Statement

The original contributions presented in the study are included in the article/Supplementary Material, further inquiries can be directed to the corresponding author/s.

## Author Contributions

MC and ZZ are the guarantor of the article. ZZ and XZ designed the study. XZ wrote the manuscript. CL and SZ collected the data. ZT and NL analyzed the data. RM, ZZ, and MC revised the manuscript. All authors approved the final version.

## Conflict of Interest

The authors declare that the research was conducted in the absence of any commercial or financial relationships that could be construed as a potential conflict of interest.
